# Management of coccydynia in the absence of X-ray evidence: Case report

**DOI:** 10.1016/j.ijscr.2018.11.043

**Published:** 2018-11-24

**Authors:** Samantha Dayawansa, David Garrett, Marcus Wong, Jason H. Huang

**Affiliations:** Department of Neurosurgery, Baylor Scott and White Health, Temple, TX, USA

**Keywords:** Coccydynia, Coccygodynia, Coccygectomy, Coccyx, Spine

## Abstract

**Background and importance:**

Coccydynia is a rare pain syndrome due to trauma to the coccyx that is diagnosed through history, physical, and sitting/standing lateral plain film radiographs. Coccydynia is typically managed conservatively with specialized cushions, NSAIDS, and physical therapy. In cases refractory to nonsurgical management, coccygectomy has a high success rate for pain reduction.

**Clinical presentation:**

Our patient had coccygeal pain for three years and endorsed a mobile fragment that was not highlighted on plain film radiographs. After evaluation with MRI and CT, a coccygeal source of their pain was highlighted and partial coccygectomy was performed.

**Conclusion:**

Post-operatively, patient endorsed major improvement in her pain at follow up. In a case of suspected coccydynia where initial imaging is inconclusive but clinical suspicion is very high, higher level imaging such as MRI or CT can reveal radiographic findings of coccydynia. MRI and CT can play a role in the diagnosis and treatment of coccydynia in the absence of x-ray evidence.

## Highlights

• The current methodology of diagnosing coccydynia revolves around sitting and standing lateral x-rays.
• If the initial imaging is negative, it can create diagnostic confusion and leave a patient without a proper diagnosis, especially in this relatively rare pain syndrome.
• Patient presented to us after failing conservative management for pain relief.
• With astute clinical suspicion and proper physical exam, high level imaging was considered after initial radiographs returned without confirmation.
• The etiology was revealed with CT and MRI, which lead to the successful surgical treatment of coccydynia and major resolution of their pain.

## 1 Background and importance

Coccydynia is a rare pain syndrome of the coccyx due to trauma or repetitive microtrauma. Often times, coccydynia is undiagnosed for many years as the pain is misattributed to lumbar stenosis or disk degeneration. It has a female-to-male ratio of 5:1, and has been reported to be more common in the overweight [1,2]. The coccyx contains four or five bones and is attached cranially to the os sacrum by the sacrococcygeal joint. It has important anatomical and physiological relationships with the fifth sacral and coccygeal nerve roots, the terminal sympathetic plexus, the filum terminale and pelvic floor musculature [3]. In addition, a rudimentary intervertebral disc may exist between Co1 and Co2, existing as a potential source of pain after trauma. Clinically, the common presentation is pain around the coccyx, worsened by sitting and rising from a seated position [4]. Uncommonly, pain can occur with defecation or intercourse. Treatment typically involves specialized cushions, oral analgesics or physical therapy. Increased pain may be managed with steroid injections or manual manipulation. In cases refractory to nonsurgical management, coccygectomy is a straightforward and successful treatment. The diagnostic work up typically includes lateral standing and sitting plain radiographs, which often show subluxation or hypermobility with greater than 25°. While diagnosis can often be done through physical examination, an unremarkable initial radiograph series can mislead a physician into pursuing an alternative pathology. Here we report a patient with coccydynia that was initially negative on x-ray, thus living with coccydynia for many years but later confirmed on CT and MRI and underwent coccygectomy, representing a role for higher level imaging when clinical suspicion is high.

## 2 Clinical presentation

**Fig. 1:**
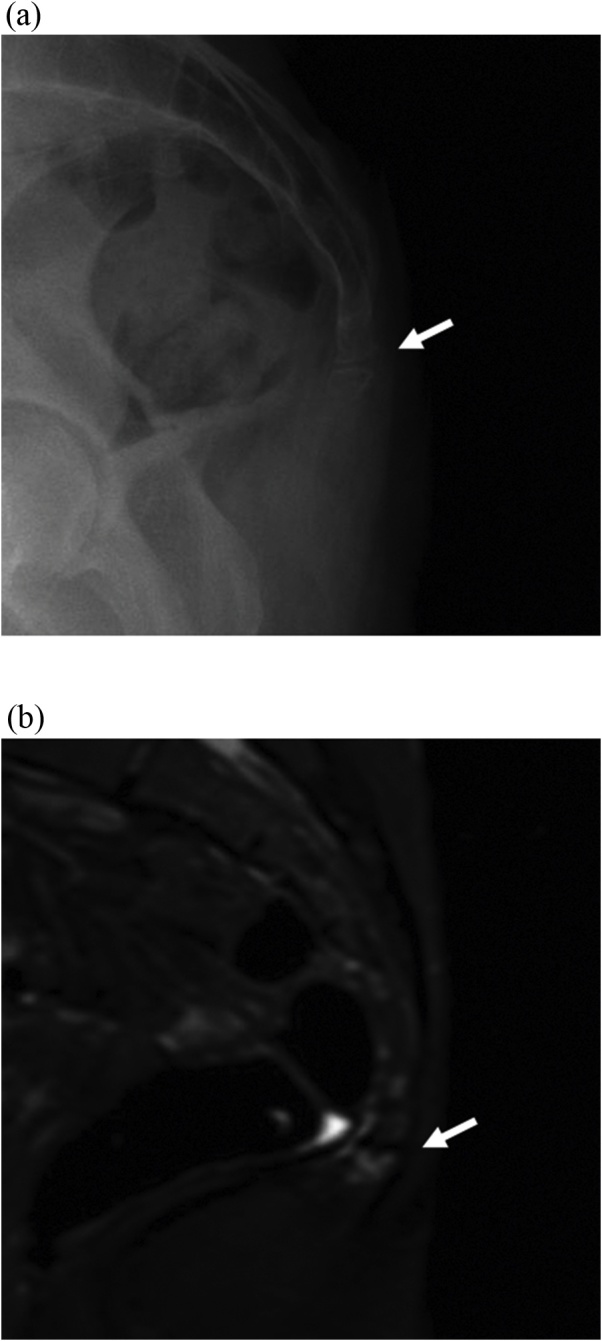
(a) Initial lateral standing coccyx x-ray demonstrating no displacement. White arrow: disruption of coccyx with inline fracture appearing as normal anatomy. (b) MRI coccyx showing marrow edema (white arrow).

**Fig. 2:**
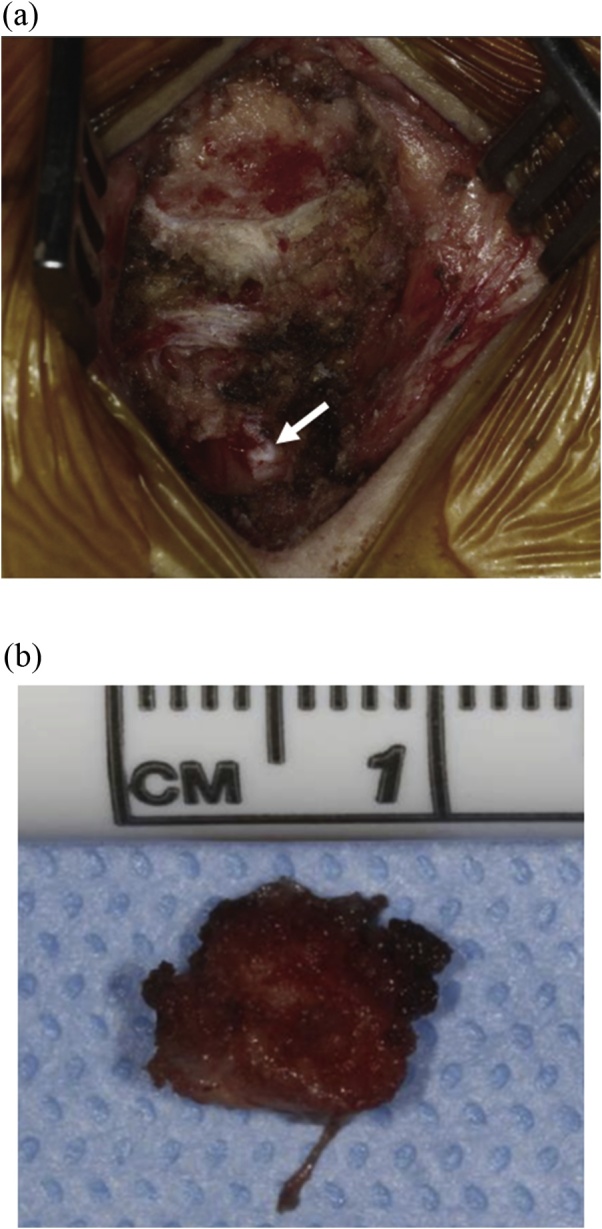
(a) Initial posterior dissection of the coccyx with free space left by removal of free fragment from previous fracture (white arrow). (b) Close up of removed free fragment.

A 28 year old female presented to clinic with a 3 year history severe non radiating pain in her “tailbone” exacerbated with sitting for more than a few minutes and relieved by standing. She recalls falling backwards while moving heavy furniture and having the pain ever since. She endorses a mobile portion of her distal coccyx, movement of which incites pain. Lateral sitting and standing x-rays in the past and during this evaluation were reported to have no fractures (Fig. 1a). Due to the clinical picture, a MRI coccyx was obtained, which revealed increased marrow edema consistent with stress reaction from healing fracture or contusion (Fig. 1b). No discrete fracture was found in any study. Patient agreed to partial coccygectomy which was performed a similar technique first described by Key [5]. During the surgery, careful dissection revealed a freely mobile and unattached distal segment (Fig. 2). The free bone and an additional level were removed using Key’s technique [5].

This patient’s post-operative recovery was uneventful and the patient reported significant improvement of her pain symptoms via numerical rating scale. At two months post op, she was much improved from her pre-operative state.

## 3 Discussion

Coccydynia is usually treated conservatively, but when pain is refractory to nonsurgical management surgical intervention may be necessary. The success rate of coccygectomy has been reported between 60%–90% [[6], [7], [8]] and to date there is no significant difference in pain reduction between partial or total coccygectomy [9]. The diagnosis and management of coccydynia is relatively straightforward, making sure to exclude alternative etiologies of pain. Once a diagnosis is suspected, sitting and standing lateral x-rays usually are sufficient for radiographic diagnosis. However, on occasions where initial imaging is negative but clinical suspicion is high, higher level imaging studies should be considered as demonstrated with our patient. The patient had a fracture of her coccyx that never healed, resulting in a free fragment causing her pain. Due to in the inline position of her fragment, fracture was ruled out on x-ray (Fig. 1). However, follow-up MRI revealed acute changes consistent with fracture that was confirmed during surgery (Fig. 2).

Limitations of the case include possibility of reader error in the initial lateral x-rays, however, we feel that the images passed through many radiologists and remained undetected over many years.

## 4 Conclusion

Here we add an experience that shows coccygectomy is effective at reducing in pain levels with low complication rates and high patient satisfaction [6]. We have shown that high level imaging studies can guide diagnosis and management, especially in those patients with unremarkable initial imaging who have had multi-year pain and a clear clinical picture of coccydynia.

## Conflicts of interest

None.

## Funding source

Baylor Scott and White Department of neurosurgery.

## Ethical approval

All procedures performed in the study were in accordance with the ethical standards of the Baylor Scott and White and/or national research committee and with the 1964 Helsinki declaration and its later amendments or comparable ethical standards. All devices utilized had PMA, 510(k), or HDE approvals and were on the shelf.

## Consent

Written informed consent was obtained from the patient for publication of this case report and accompanying images. A copy of the written consent is available for review by the Editor-in-Chief of this journal on request [10].

## Author contribution

Samantha Dayawansa: Data collection, Preparation of manuscript.

David Garrett: Data collection, Preparation of manuscript.

Jason Huang: Preparation of manuscript, Supervision.

Marcus Wong: Preparation of manuscript.

Case Study: Our institution does not require consent for 2 case reports. We have met the institution guidelines to report the findings.

## Registration of research studies

No applicable.

## Guarantor

Jason H. Huang, MD, FAANS, FACS.

## Provenance and peer review

Not commissioned, externally peer reviewed.
